# Efficacy of regional cooling + oral dexamethasone for primary prevention of hand-foot syndrome associated with pegylated liposomal doxorubicin

**DOI:** 10.1007/s00520-023-07718-2

**Published:** 2023-04-19

**Authors:** Katsuhiko Nara, Ayumi Taguchi, Takehito Yamamoto, Tetsushi Tsuruga, Yuri Tojima, Yuichiro Miyamoto, Michihiro Tanikawa, Kenbun Sone, Mayuyo Mori, Tappei Takada, Hiroshi Suzuki, Yutaka Osuga

**Affiliations:** 1 Department of Pharmacy, The University of Tokyo Hospital, Tokyo, Japan; 2 Department of Obstetrics and Gynecology, The University of Tokyo, 7-3-1 Hongo, Bunkyo-Ku, Tokyo, 113-8655 Japan; 3 The Education Center for Clinical Pharmacy, Graduate School of Pharmaceutical Sciences, The University of Tokyo, Tokyo, Japan

**Keywords:** Pegylated liposomal doxorubicin, Hand-foot syndrome, Oral dexamethasone, Regional cooling, Primary prophylaxis

## Abstract

**Purpose:**

Pegylated liposomal doxorubicin (PLD)-induced hand-foot syndrome (HFS) frequently lowers the quality of life of ovarian cancer patients. Wrist and ankle cooling, having a limited preventive effect, has been the commonest supportive HFS care. In this study, we retrospectively assessed the primary preventive effect of a combination of regional cooling and oral dexamethasone therapy (cooling + oral Dex) on HFS.

**Methods:**

This study is a single-arm retrospective, observational study. Recurrent ovarian cancer patients were administered PLD ± bevacizumab. We retrospectively examined the efficacy of hands and feet cooling (from the start of PLD to the end) + oral Dex (day 1–5: 8 mg/day, day 6, 7: 4 mg/day) for primary HFS prevention.

**Results:**

This study included 74 patients. The initial dose of PLD was 50 mg/m^2^ and 40 mg/m^2^ for 32 (43.2%) and 42 (56.8%) patients, respectively. HFS of Grade ≥ 2 and Grade ≥ 3 developed in five (6.8%) and one (1.4%) patient(s), respectively. The incidence of ≥ Grade 2 and ≥ Grade 3 HFS was much lower than those reported in previous studies. Dose reduction was required in 13 patients (17.6%) mainly because of neutropenia or mucositis; there was no HFS-induced dose reduction. Meanwhile, PLD therapy was discontinued mainly because of interstitial pneumonia (4 patients) and HFS (one patient).

**Conclusions:**

We demonstrated the efficacy of regional cooling and oral Dex for primary prevention of PLD-induced HFS. Although future prospective studies are needed to confirm its efficacy, this combination therapy can be considered for primary prevention of HFS in ovarian cancer patients on PLD.

## Introduction

Pegylated liposomal doxorubicin (PLD) is a polyethylene glycol (PEG)-coated liposomal doxorubicin. Polyethylene glycolation enables doxorubicin (DXR) to be delivered to tumor tissues without being consumed by macrophages [1]. In addition, liposomal formulation changes the drug delivery system and enhances the antitumor effect of PLD by prolonging its elimination half-life (*t*_*1/2*_), reducing the volume of distribution and selective transportation to tumor tissues [2–4].

PLD is a key drug for platinum-resistant recurrent ovarian cancer with a standard dose of 40 or 50 mg/m^2^ [5–9], and in recent years, its efficacy has improved by its combination with bevacizumab (Bev) [10]. Patients treated with PLD experience less cardiotoxicity, myelosuppression, alopecia, and vomiting than patients treated with DXR; however, they have higher risks of hand-foot syndrome (HFS) and mucositis [11–13]. The incidence of HFS has been found to be dose-dependent; the incidences of HFS ≥ Grade 2 are 10–20% and 20–50% for PLD doses of 40 mg/m^2^ and 50 mg/m^2^, respectively, and those of HFS ≥ Grade 3 are 0–5% and 10–30% for 40 mg/m^2^ and 50 mg/m^2^, respectively [5–8, 14–18]. HFS has been reported to greatly impair the quality of life (QOL) of patients with ovarian cancer; standard work and daily life activities of patients with Grade 3 HFS are largely affected [19]. Thus, preventive methods for HFS need to be established.

HFS is thought to be triggered by the following mechanisms: PLD accumulates in sweat glands in the skin [20] and generates reactive oxygen species (ROS) by interacting with copper ions. Then, ROS attack keratinocytes and trigger release of inflammatory cytokines that lead to the onset of HFS [21]. Regional cooling has been the most common strategy for the primary prevention of HFS [22–25]. Cooling of the wrists and ankles during PLD administration constricts blood vessels, thus reducing blood flow to the hands and feet. As a result, the distribution of PLD to the hands or feet is decreased and HFS can be prevented. However, the efficacy of regional cooling for prevention of HFS associated with PLD is controversial; up to 30% and 17% of patients received regional cooling reportedly developed HFS ≥ Grade 2 and ≥ Grade3, respectively [22–25], indicating that regional cooling alone is insufficient in preventing HFS associated with PLD. Meanwhile, oral steroid therapy has been recommended as a secondary preventive therapy [26]. Indeed, steroid therapy is known to reduce the levels of inflammatory cytokines associated with HFS, such as interleukin (IL)-1 and IL-6 [27].

Considering that regional cooling and oral steroid therapy prevent HFS through independent mechanisms, there is a possibility that combining both strategies may further prevent HFS efficiently. In The University of Tokyo Hospital, we have been using the combination therapy of regional cooling and oral dexamethasone (Dex) as a primary prevention of HFS since 2009. In this study, we retrospectively examined the efficacy and safety of regional cooling + oral Dex for primary prevention and compared the incidence of HFS with those reported in previous literature.

## Patients and methods

### Patients

In this retrospective, observational, single-arm study, patients who received regional cooling and 7 days of oral Dex (hereafter referred to as “cooling + Dex”) as primary prevention for HFS from the day of PLD/PLD + Bev administration for the treatment of recurrent ovarian cancer, fallopian tube cancer, or peritoneal carcinoma at The University of Tokyo Hospital from December 2009 to December 2021 were included. However, patients who had continuously been administrated steroid drugs prior to the start of PLD monotherapy or PLD + Bev therapy or those who did not complete the primary prevention with cooling + Dex were excluded.

### Treatments

PLD monotherapy consisted of PLD (40 or 50 mg/m^2^, i.v.), granisetron (3 mg, i.v.) or ramosetron (0.3 mg, i.v.) or azasetron (10 mg, i.v), and Dex (6.6 mg, i.v.) on day 1 of 28-day cycles. PLD + Bev therapy was similar to PLD monotherapy except that Bev (15 mg/m^2^, i.v.) was added on day 1. Primary prevention with cooling + Dex consisted of regional cooling with ice packs (from wrists to hands, and ankles to feet) during the administration of PLD (90 min) and oral Dex for 7 days after PLD administration (8 mg/day on days 1–5, and 4 mg/day on days 6 and 7). During the administration of PLD (90 min), ice packs were not replaced. Because the *t*_*1/2*_ of PLD is long (approximately 80 h) and the severity of HFS has been reportedly associated with *t*_*1/2*_ [28], we set the treatment duration of oral Dex at 7 days (approximately twice the *t*_*1/2*_ of PLD) until the blood concentration of PLD was sufficiently reduced.

### Data collection and definition

Demographic and clinical data collected from the medical records of the patients included the following: age, sex, body mass index (BMI), performance status (PS), types of primary cancer, pathological diagnosis, cancer staging based on the International Federation of Gynecology and Obstetrics (FIGO) criteria, number of previous chemotherapy, initial PLD dose, number of cycles (PLD monotherapy or PLD + Bev therapy), supportive care, and laboratory data (aspartate aminotransferase (AST), alanine aminotransferase (ALT), serum total bilirubin (T-Bil), serum creatinine (Cre), creatinine clearance (CCr), c-reactive protein (CRP), white blood cell count (WBC), absolute neutrophil count (ANC), platelet count (PLT), hemoglobin (Hb), and absolute lymphocyte count (ALC)) before PLD monotherapy or PLD + Bev therapy. We also investigated the severity of adverse events (AEs) including HFS. The severity of AEs were retrospectively evaluated according to the National Cancer Institute Common Toxicity Criteria for Adverse Events (CTCAE) version 5.0. In addition, to investigate the association between the numbers of chemotherapy cycles and the severities of HFS, the number of patients who experienced HFS > Grade 1 was counted at each chemotherapy cycle. Further, we investigated the incidences of and reasons for dose reduction/discontinuation as informed by the attending physicians in the medical records.

## Results

### Patient characteristics

**Fig. 1:**
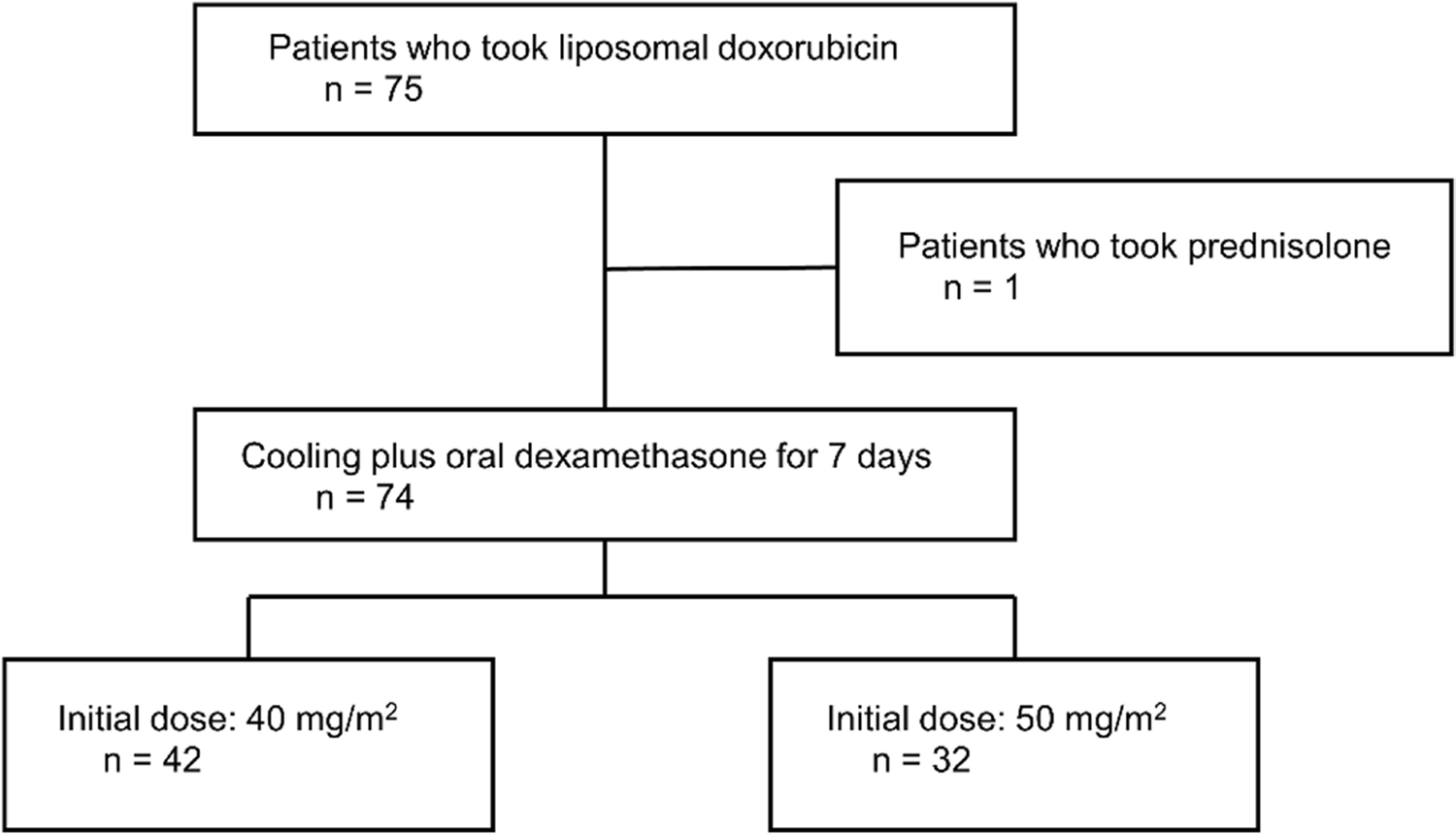
Flow chart of patient enrollment

Among the 75 patients who received PLD monotherapy or PLD + Bev therapy, one patient, receiving prednisolone for comorbidities, was excluded, and the remaining 74 patients were included in the following analyses (Fig. 1). All patients included in this study have completed the entire duration of “cooling + Dex” prophylaxis during PLD administration.

Table 1 shows the characteristics of the 74 patients. The initial dose of PLD was 50 mg/m^2^ for 32 patients (43.2%) and 40 mg/m^2^ for 42 patients (56.8%). Bev was concomitantly administrated to 16 patients (21.6%). Approximately half of the patients had received at least four chemotherapy regimens prior to PLD monotherapy or PLD + Bev therapy. No patients had any residual HFS ≥ G1 at the time of PLD initiation.

**Table 1:** Patients’ background

	All patients (*n* = 74)	Initial dose of PLD	
		40 mg/m^2^ (*n* = 42)	50 mg/m^2^ (*n* = 32)
Age [year], median (range)	59.1 (37.6–81.9)	55.4 (37.6–81.9)	59.8 (39.4–77.7)
BMI [kg/m^2^], median (range)	22.2 (15.5–34.5)	22.5 (16.4–32.3)	22.0 (15.5–34.5)
Performance status, n (%)			
0	43 (58.1)	27 (64.3)	16 (50.0)
1	31 (41.9)	15 (35.7)	16 (50.0)
Primary cancer, n (%)			
Ovarian cancer	65 (87.8)	38 (90.5)	27 (84.4)
Fallopian tube cancer	3 (4.1)	2 (4.8)	1 (3.1)
Peritoneal cancer	6 (8.1)	2 (4.8)	4 (12.5)
Tumor histology, n (%)			
Serous	42 (56.8)	25 (59.5)	17 (53.1)
Endometrioid	11 (14.9)	8 (19.0)	3 (9.4)
Clear cell	8 (10.8)	3 (7.1)	5 (15.6)
Mucinous	3 (4.1)	1 (2.4)	2 (6.3)
Other	10 (13.5)	5 (11.9)	5 (15.6)
Stage at diagnosis, n (%)			
I	7 (9.5)	4 (9.5)	3 (9.4)
II	3 (4.1)	0 (0.0)	3 (9.4)
III	40 (54.1)	28 (66.7)	12 (37.5)
IV	22 (29.7)	10 (23.8)	12 (37.5)
Unknown	2 (2.7)	0 (0.0)	2 (6.3)
Previous chemotherapy, n (%)			
1 regimen	10 (13.5)	6 (14.3)	4 (12.5)
2 regimens	18 (24.3)	8 (19.0)	10 (31.3)
3 regimens	10 (13.5)	4 (9.5)	6 (18.8)
≥ 4 regimens	36 (48.6)	24 (57.1)	12 (37.5)
No. of chemotherapy cycles, median (range)	3 (1–22)	3 (1–15)	4 (1–22)
Bevacizumab combination, n (%)	16 (21.6)	15 (35.7)	1 (3.1)
Supportive care, n (%)			
Moisturizing agent	18 (24.3)	13 (31.0)	5 (15.6)
5-HT_3_ blocker			
Granisetron	53 (71.6)	41 (97.6)	12 (37.5)
Ramosetron	16 (21.6)	1 (2.4)	15 (46.9)
Azasetron	5 (6.8)	0 (0.0)	5 (15.6)
Laboratory data, median (range)			
AST [U/L]	21 (9–72)	22 (15–72)	21 (9–42)
ALT [U/L]	15 (5–79)	15 (6–79)	15 (5–33)
T-Bil [mg/dL]	0.5 (0.3–1.4)	0.5 (0.3–1.0)	0.4 (0.3–1.4)
Cre [mg/dL]	0.65 (0.43–0.95)	0.62 (0.47–0.94)	0.67 (0.43–0.95)
Ccr [mL/min]	80.6 (28.9–161.7)	87.4 (28.9–161.7)	71.8 (34.6–138.9)
CRP [mg/dL]	0.41 (0.02–9.50)	0.26 (0.02–6.00)	0.77 (0.02–9.50)
WBC [cells/μL]	5150 (2300–10900)	5250 (2300–10900)	4950 (2300–9600)
ANC [cells/μL]	3150 (1100–7900)	3250 (1100–7900)	3000 (1100–7000)
Plt [10^4^ cells/μL]	27.4 (7.6–60.6)	27.2 (11.8–43.8)	30.2 (7.6–60.6)
Hb [g/dL]	11.2 (7.4–14.7)	11.3 (7.4–14.7)	11.0 (7.4–13.3)
ALC [cells/μL]	1250 (500–3300)	1300 (500–3300)	1200 (600–2100)

PLD, pegylated liposomal doxorubicin; 5-HT_3_, 5-hydroxytryptamine 3 receptor; BMI, body mass index; AST, aspartate transaminase; ALT, alanine transaminase; T-Bil, total bilirubin; Cre, serum creatinine; Ccr, creatinine clearance; CRP, C- reactive protein; WBC, white blood cell count; ANC, absolute neutrophil counts; Plt, platelet count; Hb, hemoglobin; ALC, absolute lymphocyte counts

### Adverse events and dose reduction/discontinuation

The incidences of AEs associated with PLD monotherapy or PLD + Bev therapy are summarized in Table 2. Overall, patients receiving 50 mg/m^2^ of PLD tended to experience more severe AEs than those receiving 40 mg/m^2^ of PLD. HFS ≥ Grade 2 was observed in three (9.4%) and two (4.8%) patients in the 50 mg/m^2^ and 40 mg/m^2^ groups, respectively. In total, only one patient (1.4%) experienced HFS ≥ Grade 3. Regarding non-hematological toxicities, mucositis was most frequently observed; twenty-seven (36.5%) and five (6.8%) patients experienced ≥ Grade 2 and ≥ Grade 3 mucositis, respectively, and none experienced ≥ Grade 3 nausea and vomiting.

**Table 2:** Adverse events of PLD ± Bev

Adverse events	Initial dose of PLD									
	40 mg/m^2^ (*n* = 42)					50 mg/m^2^ (*n* = 32)				
	Grade 2	Grade 3	Grade 4	≥ Grade 2	≥ Grade 3	Grade 2	Grade 3	Grade 4	≥ Grade 2	≥ Grade 3
Neutropenia, n (%)	15 (35.7)	8 (19.0)	4 (9.5)	27 (64.3)	12 (28.6)	7 (21.9)	10 (31.3)	7 (21.9)	24 (75.0)	17 (53.1)
Febrile neutropenia, n (%)	0 (0.0)	1 (2.4)	0 (0.0)	1 (2.4)	1 (2.4)	0 (0.0)	5 (15.6)	0 (0.0)	5 (15.6)	5 (15.6)
Thrombocytopenia, n (%)	3 (7.1)	0 (0.0)	1 (2.4)	4 (9.5)	1 (2.4)	1 (3.1)	2 (6.3)	0 (0.0)	3 (9.4)	2 (6.3)
Vomiting, n (%)	0 (0.0)	0 (0.0)	0 (0.0)	0 (0.0)	0 (0.0)	0 (0.0)	0 (0.0)	0 (0.0)	0 (0.0)	0 (0.0)
Nausea, n (%)	2 (4.8)	0 (0.0)	0 (0.0)	2 (4.8)	0 (0.0)	6 (18.8)	0 (0.0)	0 (0.0)	6 (18.8)	0 (0.0)
Mucositis, n (%)	7 (16.7)	2 (4.8)	0 (0.0)	9 (21.4)	2 (4.8)	15 (46.9)	3 (9.4)	0 (0.0)	18 (56.3)	3 (9.4)
Diarrhea, n (%)	0 (0.0)	0 (0.0)	0 (0.0)	0 (0.0)	0 (0.0)	0 (0.0)	0 (0.0)	0 (0.0)	0 (0.0)	0 (0.0)
Hand-foot syndrome, n (%)	2 (4.8)	0 (0.0)	0 (0.0)	2 (4.8)	0 (0.0)	2 (6.3)	1 (3.1)	0 (0.0)	3 (9.4)	1 (3.1)
Interstitial pneumonia, n (%)	1 (2.4)	1 (2.4)	0 (0.0)	2 (4.8)	1 (2.4)	0 (0.0)	1 (3.1)	0 (0.0)	2 (6.3)	2 (6.3)
Acute infusion reaction, n (%)	4 (9.5)	0 (0.0)	0 (0.0)	4 (9.5)	0 (0.0)	0 (0.0)	0 (0.0)	0 (0.0)	0 (0.0)	0 (0.0)

PLD, pegylated liposomal doxorubicin

Table 3 shows the incidences of dose reduction and discontinuation. In total, 13 (17.6%) patients needed dose reduction and 11 (14.9%) patients discontinued PLD therapy due to severe AEs (Table 3). Dose reduction was more frequent at 50 mg/m^2^; dose reduction was required in two (4.8%) and eleven patients (34.4%) receiving 40 mg/m^2^ and 50 mg/m^2^ of PLD, respectively. The most common AEs that led to dose reduction were mucositis and neutropenia. However, no patient required dose reduction due to HFS. In total, 11 patients abandoned PLD therapy due to severe AEs. The most common AE that led to discontinuation of PLD was interstitial pneumonia followed by mucositis. There was only one patient (1.4%) who needed to discontinue the chemotherapy due to HFS (Table 3).

**Table 3:** Incidences and reasons of dose reduction/discontinuation

	Initial dose of PLD		
	40 mg/m^2^ (*n* = 42)	50 mg/m^2^ (*n* = 32)	Total (*n* = 74)
Dose reduction of PLD, n (%)	2 (4.8)	11 (34.4)	13 (17.6)
Cycles of dose reduction, median (range)	3 (2–4)	2 (2–4)	2 (2–4)
Reason of dose reduction			
Mucositis, n (%)	1 (2.4)	3 (9.4)	4 (9.5)
Neutropenia, n (%)	0 (0.0)	3 (9.4)	3 (4.1)
Anemia, n (%)	0 (0.0)	2 (6.3)	2 (2.7)
Others, n (%)	1 (2.4)	3 (9.4)	4 (9.5)
Discontinuation of PLD, n (%)	42 (100.0)	32 (100.0)	74 (100.0)
Cycles of discontinuation, median (range)	3 (1–15)	4 (1–22)	3 (1–22)
Reason of discontinuation			
Progression disease, n (%)	35 (83.3)	28 (87.5)	63 (85.1)
Adverse events, n (%)	7 (16.7)	4 (12.5)	11 (14.9)
Interstitial pneumonia, n (%)	2 (4.8)	2 (6.3)	4 (9.5)
Mucositis, n (%)	2 (4.8)	1 (3.1)	3 (4.1)
Hand-foot syndrome, n (%)	0 (0.0)	1 (3.1)	1 (1.4)
Other adverse events, n (%)	3 (7.1)	0 (0.0)	3 (4.1)

PLD, pegylated liposomal doxorubicin

### Association between the number of chemotherapy cycle and the severity of HFS

**Fig. 2:**
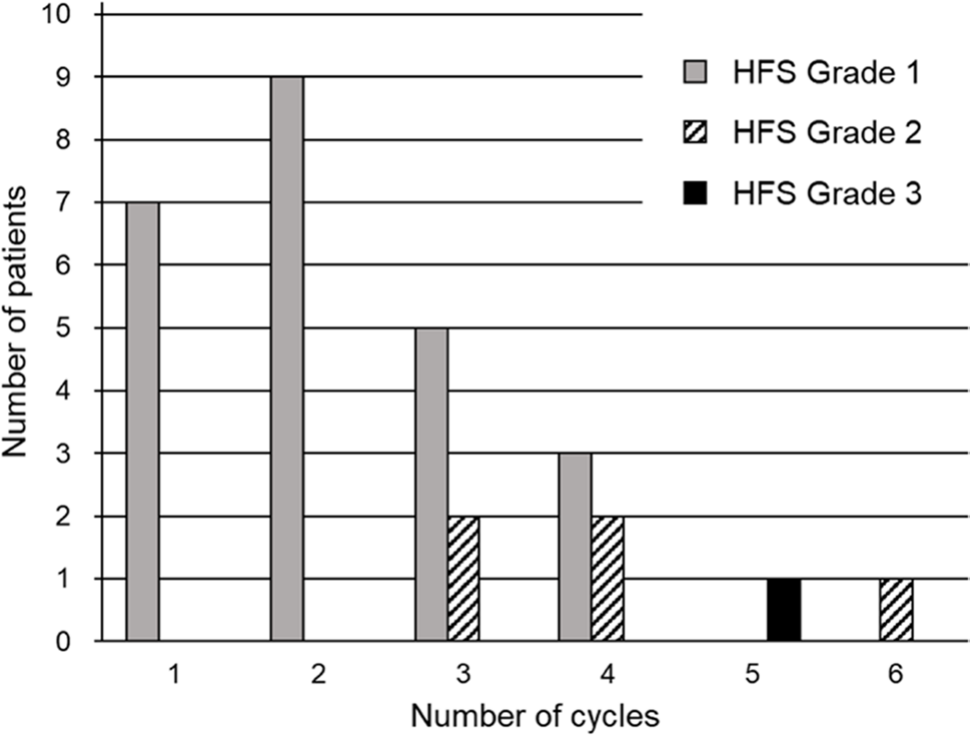
HFS incidence during multiple chemotherapy cycles

Figure 2 demonstrates the number of patients who experienced HFS ≥ Grade 1 at each chemotherapy cycle. As shown in Fig. 2, although the number of patients experiencing HFS tended to decrease upon increase in number of cycles, HFS severity tended to worsen upon increase in number of cycles.

## Discussion

This study firstly demonstrated the efficacy of combining regional cooling and oral Dex for primary prevention of PLD-induced HFS. The combination therapy of regional cooling and oral Dex led to a lower incidence of HFS (HFS ≥ Grade 2, 7.6% and HFS ≥ Grade 3, 1.5%) than that reported with regional cooling [22–25]. The results of this study support the usefulness of cooling + oral Dex as primary prevention for HFS in ovarian cancer patients receiving PLD monotherapy or PLD + Bev therapy.

In the current study, cooling + Dex therapy demonstrated a sufficient prevention of HFS associated to PLD regardless of the PLD dose; HFS ≥ Grade 2 occurred in 5.9% and 9.4% of patients receiving 40 mg/m^2^ and 50 mg/m^2^ of PLD, respectively, and HFS ≥ Grade 3 occurred in 0.0% and 3.1% of patients receiving 40 mg/m^2^ and 50 mg/m^2^ of PLD, respectively. Considering that the preventive efficacy of regional cooling alone was controversial in previous reports (up to 30% and 17% of incidence for HFS ≥ Grade 2 and ≥ Grade 3, respectively) [22–25], these results indicate that at least part of the observed preferable preventive efficacy is attributed to the addition of 7 days of oral Dex to the regional cooling. Previously, we had used a combination therapy of regional cooling and 2 days of oral Dex (8 mg/day on days 1 and 2) for six patients prior to the current postulated protocol. Among these six patients, two (33%) each experienced Grade 2 and Grade 3 HFS (unpublished data), indicating that 2 days of oral Dex might be less effective in preventing HFS compared with 7 days of oral Dex. These observations seem reasonable considering the long *t*_*1/2*_ of PLD (approximately 80 h). Although regional cooling alone is effective in preventing HFS by reducing the distribution of PLD into hands and feet, PLD can remain in the body for a long period and induce inflammatory reaction. Therefore, it would be beneficial to maintain the anti-inflammatory effect by administrating Dex for a period well beyond the *t*_*1/2*_ of PLD.

Regarding AEs other than HFS, previous prospective studies have reported nausea + vomiting ≥ Grade 2 and ≥ Grade 3 at frequencies of 15–20% and 5–10% [5, 7, 8, 14–16], respectively, whereas in our study, the incidences of nausea + vomiting ≥ Grade 2 and ≥ Grade 3 were 10.8% (8/74) and 0.0% (0/74), respectively (Table 2): the incidence of nausea + vomiting tended to be lower than those previously reported in the literature. This observation seems reasonable considering that Dex is an established anti-emetic agent and is used with many cancer chemotherapies. In contrast, in our study population, mucositis ≥ Grade 2 and Grade 3 occurred in 36.5% (22/74) and 6.8% (5/74) of patients, respectively. Particularly, in patients receiving 50 mg/m^2^ of PLD, mucositis ≥ Grade 2 occurred in 56.3% (18/32). Consequently, mucositis was the leading cause of dose reduction instead of HFS. The incidences of Grade 2 and 3 mucositis were relatively higher than those previously reported in the literature (incidences of mucositis ≥ Grade 2 and Grade 3 were 15–30% and 5–10%, respectively) [5, 7, 8, 14–16].

The high incidence of mucositis might be attributed to the ambivalent effect of oral Dex. Similar to HFS, mucositis is considered to be caused by free radicals and inflammatory cytokines from anticancer agents that destroy the organization of the oral mucosa [29]. However, different from HFS, secondary infection with oral bacteria, which can be exacerbated by chemotherapy-induced neutropenia, augments mucositis [29]. Oral Dex may reduce the production of free radicals, inflammatory cytokines, and subsequent mucosal injuries [27]; however, bacterial infection may be worsened due to steroid therapy. In addition, steroid therapy sometimes causes not only mucositis but also other AEs including insomnia and hyperglycemia. Most especially, AEs are more likely to occur when patients take a long course of oral steroids. Therefore, optimal duration of oral Dex should be further investigated in future prospective studies. In addition, oral pyridoxine, which has been reported to prevent PLD-induced HFS [30], may be used to reduce the risk of mucositis. A phase II study reported low incidence of mucositis (2.8% for both Grades 1–2 and 3–4) in patients receiving pyridoxine (300 mg/day) during PLD + paclitaxel therapy [31]. Considering the relatively nontoxic characteristics of pyridoxine, adding oral pyridoxine to cooling + Dex therapy may reduce the risk of mucositis without decreasing the protective effect against HFS. Hence, the efficacy of pyridoxine + cooling + Dex therapy seems to be worth investigating in future clinical studies.

This study has several limitations. First, this is a retrospective and observational study. There might be some missing data, and the assessment of AEs can vary between physicians. In addition, it was difficult to confirm how strictly regional cooling was performed on each patient each time. Those limitations may have led to an underestimation of the incidence of AEs, making it difficult to accurately assess the preventive effect of the combination therapy. Second, this study is not a case–control study. We only compared the incidence of HFS in our cohort with those in previous reports. Direct comparison is needed to confirm the preventive effect against HFS. Third, in this study, AEs of seven-day oral Dex were not sufficiently assessed. Oral steroids may cause hyperglycemia, insomnia, osteopenia, and bacterial infection. Further prospective controlled studies to assess the efficacy and safety of oral Dex as primary prevention of HFS are warranted. Despite these limitations, this study is the first to demonstrate the preventive effect of regional cooling + oral Dex on PLD-induced HFS.

In conclusion, we demonstrated the efficacy of the combination therapy of regional cooling and oral Dex for primary prevention of PLD-induced HFS. Although future prospective studies are needed to confirm its efficacy, this combination therapy can be considered for primary prevention of HFS in ovarian cancer patients receiving PLD.

## Data Availability

The data that support the findings of this study are available upon request to the corresponding author. The data are not publicly available due to privacy or ethical restrictions.
